# Survival, neurocognitive function, and health-related quality of life outcomes after rituximab—methotrexate, BCNU, teniposide, and prednisolone for primary CNS lymphoma: Final results of the HOVON 105/ALLG NHL 24 study

**DOI:** 10.1093/neuonc/noad224

**Published:** 2023-12-01

**Authors:** Jacoline E C Bromberg, Samar Issa, Bronno van der Holt, Matthijs van der Meulen, Linda Dirven, Monique C Minnema, Tatjana Seute, Marc Durian, Gavin Cull, Marjolein W M van der Poel, Wendy B C Stevens, Josee M Zijlstra, Dieta Brandsma, Marcel Nijland, Kylie D Mason, Aart Beeker, Martine C J Abrahamse-Testroote, Martin J van den Bent, Daphne de Jong, Jeanette K Doorduijn

**Affiliations:** Department of Neuro-Oncology, Erasmus MC Cancer Institute, Rotterdam, The Netherlands; Department of Hematology, Middlemore Hospital, Auckland, New Zealand; HOVON Foundation, Rotterdam, The Netherlands; Department of Hematology, Erasmus MC Cancer Institute, Rotterdam, The Netherlands; Department of Neurology, Medisch Spectrum Twente, Enschede, The Netherlands; Department of Neurology, Leiden University Medical Center, Leiden, The Netherlands; Department of Neurology, Haaglanden Medical Center, The Hague, The Netherlands; Department of Hematology, University Medical Center Utrecht, The Netherlands; Department of Neurology, University Medical Center, Utrecht, The Netherlands; Department of Hematology, ETZ Hospital, Tilburg, The Netherlands; Sir Charles Gairdner Hospital and PathWest Laboratory Medicine, Nedlands, Western Australia, Australia; Department of Hematology, University of Western Australia, Crawley, Western Australia, Australia; Department of Internal Medicine, Division of Hematology, GROW School for Oncology and Developmental Biology, Maastricht University Medical Center, Maastricht, The Netherlands; Department of Hematology, Radboud University Medical Center, Nijmegen, The Netherlands; Department of Hematology, Amsterdam UMC, VUMC, Amsterdam, The Netherlands; Department of Neuro-Oncology, Netherlands Cancer Institute, Amsterdam, The Netherlands; Department of Hematology, UMCG, Groningen, The Netherlands; Department of Hematology, Royal Melbourne Hospital, Melbourne, Australia; Department of Hematology, Spaarne Gasthuis, Haarlem, The Netherlands; HOVON Foundation, Rotterdam, The Netherlands; Department of Neuro-Oncology, Erasmus MC Cancer Institute, Rotterdam, The Netherlands; Department of Pathology and HOVON Pathology, Facility and Biobank, Amsterdam UMC, VUMC, Amsterdam, The Netherlands; Department of Hematology, Erasmus MC Cancer Institute, Rotterdam, The Netherlands

**Keywords:** health-related quality of life, neurocognitive function, PCNSL, rituximab, treatment

## Abstract

**Background:**

Studies on the efficacy of rituximab in primary CNS lymphoma (PCNSL) reported conflicting results. Our international randomized phase 3 study showed that the addition of rituximab to high-dose methotrexate, BCNU, teniposide, and prednisolone (MBVP) in PCNSL was not efficacious in the short term. Here we present long-term results after a median follow-up of 82.3 months.

**Methods:**

One hundred and ninety-nine eligible newly diagnosed, nonimmunocompromised patients with PCNSL aged 18–70 years with WHO performance status 0–3 was randomized between treatment with MBVP chemotherapy with or without rituximab, followed by high-dose cytarabine consolidation in responding patients, and reduced-dose WBRT in patients aged ≤ 60 years. Event-free survival was the primary endpoint. Overall survival rate, neurocognitive functioning (NCF), and health-related quality of life (HRQoL) were additionally assessed, with the IPCG test battery, EORTC QLQ-C30 and QLQ-BN20 questionnaires, respectively.

**Results:**

For event-free survival, the hazard ratio was 0.85, 95% CI 0.61–1.18, *P* = .33. Overall survival rate at 5 years for MBVP and R-MBVP was 49% (39–59) and 53% (43–63) respectively. In total, 64 patients died in the MBVP arm and 55 in the R-MBVP arm, of which 69% were due to PCNSL. At the group level, all domains of NCF and HRQoL improved to a clinically relevant extent after treatment initiation, and remained stable thereafter up to 60 months of follow-up, except for motor speed which deteriorated between 24 and 60 months. Although fatigue improved initially, high levels persisted in the long term.

**Conclusions:**

Long-term follow-up confirms the lack of added value of rituximab in addition to MBVP and HD-cytarabine for PCNSL.

**Key Points**

- No effect of rituximab on overall survival in PCNSL patients treated with MBVP and HD-Ara-C.
- Recurrences continue to occur during at least 10 years of follow-up.
- Clinically relevant fatigue persists for years in a significant proportion of patients.

**Importance of the Study**

Primary CNS lymphoma (PCNSL) is a rare disease in which the large majority of published papers concern retrospective studies with inherent biases. Little prospective, long-term follow-up data is available both regarding survival data and, especially, neurocognitive functioning and health-related quality of life data. Moreover, the efficacy of rituximab in PCNSL is still unclear.

In our prospective, randomized, phase 3 study we showed that, when added to effective polychemotherapy such as the MBVP regimen, rituximab does not significantly impact survival outcomes including overall survival. Secondly, we show that recurrences continue to occur in the long term albeit less frequently than in the first 3 years, at least in patients not consolidated with myeloablative chemotherapy and ASCT. Last but not least we show that, in the long term, neurocognitive functioning is preserved in a majority of patients but clinically relevant fatigue persists for years in a significant proportion of patients.

Primary central nervous system lymphoma (PCNSL) is a rare, diffuse large B-cell lymphoma with disease activity restricted to the brain, spine, leptomeninges, and eyes. The incidence of PCSNL has increased over the last decades,^1,2^ but few prospective, long-term follow-up data are available.^3–7^ The efficacy of rituximab in PCNSL is still under debate after 2 randomized studies which yielded conflicting results regarding survival outcomes.^8–10^ We previously reported initial results of the phase 3 HOVON 105/ALLG NHL 24 study, in which we randomized patients with newly diagnosed PCNSL to high-dose methotrexate (HD-MTX)-based chemotherapy with or without rituximab, followed by high-dose cytarabine, and, in patients up to 60 years old, 30 Gy whole brain radiotherapy (WBRT). After a median follow-up of 33 months, we observed negative trial results for the primary endpoint, event-free survival (EFS) (hazard ratio 1.01, 95% CI 0.70–1.43, *P* = .99).^9^ There were no differences between treatment arms for neurocognitive functioning (NCF) and health-related quality of life (HRQoL).^11,12^ Both NCF and HRQoL improved significantly, with clinically relevant improvements as compared to baseline in all selected HRQoL domains and the neurocognitive domain “motor speed.” For both NCF and HRQoL, scores remained stable up to 2 years after treatment after the initial improvement. In irradiated patients (ie patients ≤ 60 years old), NCF and HRQoL also remained stable after radiotherapy for up to 2 years after treatment. The long-term effect of adding rituximab to MBVP chemotherapy on survival is unknown. Moreover, data regarding the NCF and HRQoL in long-term PCNSL survivors are limited.^13^ Here we report long-term follow-up results on primary and secondary endpoints including overall survival (OS), NCF, and HRQoL outcomes in the HOVON 105/ALLG NHL 24 study.

## Methods

### Patients and Treatment

Between 2010 and 2016, 199 eligible immunocompetent patients aged 26–70 years (median 61) with newly diagnosed PCNSL were randomly assigned to MBVP (two 28-day cycles of HD-MTX 3 g/m^2^ on days 1 and 15, teniposide 100 mg/m^2^ on days 2 and 3, carmustine 100 mg/m^2^ on day 4, prednisolone 60 mg/m^2^ on days 1–5) with or without i.v., rituximab 375 mg/m^2^ on days 0, 7, 14, 21 in cycle 1, and days 0 and 14 in cycle 2.^2^ All responding patients subsequently received high-dose cytarabine 2 g/m^2^ consolidation twice daily on 2 consecutive days. Only patients aged ≤60 years were further consolidated with 30 Gy WBRT and, in case of residual disease, an integrated boost of 20 × 0.5 Gy on the tumor bed. All patients, or caregivers in case patients were unable to, gave written informed consent. The study was conducted in accordance with the Declaration of Helsinki and Good Clinical Practice, and approved by the Institutional Review Boards of the Erasmus MC University Medical Center, Rotterdam, The Netherlands, and the respective ethics committees of both New Zealand and Australia. Patients from centers participating in the NCF and HRQoL sub studies additionally provided consent for NCF and HRQoL evaluation at various time points.

### Outcome Measures

The response was assessed according to the International Primary CNS Lymphoma Collaborative Group (IPCG) criteria^14^ and EFS, PFS, and OS were calculated for the whole trial population from the date of inclusion into the study as previously detailed.^9^ For evaluation of NCF and HRQoL, a brief standardized test battery covering various neurocognitive domains,^15^ slightly adapted because not all tests were available in the Dutch language, (Supplementary Table 1) and the EORTC QLQ-C30^16^ and QLQ-BN 20^16,17^ questionnaires were performed at baseline, after induction and consolidation treatments, and 3, 6, 12, and 24 months after treatment. Patients from 6 of the original 13 centers participating in the NCF and HRQoL analyses, additionally were asked to complete assessments 36, 48, and/or 60 months after treatment or until progression. The primary end-point was EFS, defined as the absence of complete response (CR) or unconfirmed complete response (CRu) at the end of all protocol treatment, or relapse or death after previous CR or CRu. Secondary end-points were response rates, toxicity, progression-free survival (PFS), and OS, as well as NCF and HRQoL outcomes over time. All survival measures were calculated for the whole trial population; NCF and HRQoL analyses were performed on the survivor population.

Results of individual scores on the neurocognitive tests were transformed into *z*-scores adjusted for age, sex, and/or level of education. HRQoL scores were calculated according to the guidelines and range from 0 to 100.^18^ Compliance with NCF and HRQoL assessments at each time point was defined as the number of completed NCF tests/questionnaires at a specific time point divided by the number of evaluations expected at that time point. Specific predetermined NCF domains and HRQoL scales were selected for primary analysis and reported on; relevant NCF domains were attention/executive functioning, information processing speed, memory, and motor speed, while global health status, role functioning, social functioning, fatigue, and motor dysfunction were considered relevant HRQoL outcomes.

Changes over time in NCF at group level and at individual patient level were determined for each preselected domain with changes in *z*-scores of ≥1 SD and changes in HRQoL scores of ≥10 points were considered clinically relevant.^19–23^ Patients with NCF/HRQoL data 36, 48, and/or 60 months after treatment were eligible for this analysis; for evaluating changes from baseline, only patients with both a baseline evaluation and at least one long-term evaluation were eligible. For evaluating changes after chemotherapy treatment only patients with both an “ after chemotherapy” and at least 1 long-term evaluation were eligible.

### Statistical Analysis

Descriptive statistics (eg means or medians with SD and range, respectively, or percentages) were used to describe the patient population and the NCF and HRQoL outcomes.

Analyses of survival end-points were according to the modified intention to treat principle, ie patients were analyzed according to the treatment arms they were assigned to. Patients who were initially randomized but considered to be ineligible in hindsight were excluded from all analyses. The primary endpoint EFS was evaluated using a multivariable Cox proportional hazards (PH) regression analysis with adjustment for randomization minimization factors age (≤60 vs. ≥61 years) and WHO performance status (0–1 vs. 2–3). In an exploratory fashion, the effect of various prognostic factors on the primary and secondary endpoints was assessed using univariable and multivariable logistic and Cox PH regression analyses.

For both NCF and HRQoL analyses, evaluations were analyzed on a group level (ie the magnitude of change over time) and an individual patient level (ie responder analysis).^24^ All included patients in this long-term follow-up assessment were analyzed as 1 group, and not separately by treatment arm since no differences were found between the groups in the initial NCF and HRQoL analyses on the short-term and the number of patients per treatment arm was too small at several time points to allow meaningful comparison.^11,12^ Due to the limited number of patients in this survivor population, all results were only analyzed descriptively. On a group level, scores over time for all preselected NCF and HRQoL domains were described and interpreted in terms of clinically relevant changes. On an individual level, the percentage of responders was calculated, defined as having a clinically relevant improvement in scores at each assessment during the 60-month follow-up period as compared to baseline. For the statistical analyses Stata version 16.1 was used. See previous reports for further details.^9,11,12^

## Results

At database lock on February 3, 2022, 80 of 199 included patients were alive and had a median follow-up of 82.3 months. The main clinical characteristics and outcomes are shown in Table 1.

**Table 1.** Main Characteristics and Outcome of Eligible Patients

	MBVP (*n* = 100)	R-MBVP (*n* = 99)	
Median age years (range)	61 (26–70)	61 (37–70)	
≥61	53 (53%)	52 (53%)	
Sex: women	39 (39%)	51 (52%)	
Performance status			
WHO 0	20 (20%)	23 (23%)	
WHO 1	51 (51%)	50 (51%)	
WHO 2	18 (18%)	16 (16%)	
WHO 3	11 (11%)	10 (10%)	
RTOG neurologic function			
0	5 (5%)	14 (14%)	
1	25 (25%)	26 (26%)	
2	19 (19%)	13 (13%)	
3	28 (28%)	21 (21%)	
4	8 (8%)	15 (15%)	
Unknown	15 (15%)	10 (10%)	
Pathology DLBCL	99 (99%)	97 (98%)	
Multifocal disease	51 (51%)	46 (46%)	
CSF involvement yes	16 (16%)	18 (18%)	
No	53 (53%)	52 (53%)	
Unknown	31 (31%)	29 (29%)	
Vitreous fluid localization	3 (3%)	8 (8%)	
LDH elevated	29 (29%)	29 (29%)	
MSKCC prognostic score			
Age ≤ 50 y	12 (12%)	16 (16%)	
Age > 50, WHO < 1	59 (59%)	63 (64%)	
Age > 50, WHO > 1	29 (29%)	20 (20%)	
			**Total**
EFS, months, median (95% CI)	10.8 (6.0–25.6)	15.5 (8.0–42.8)	14.9 (7.9–24.5)
5 y EFS (95% CI)	25% (17–34)	36% (27–46)	HR 0.85 (0.61–1.18, *P* = .33)
PFS, months, median (95% CI)	24 (10.5–28.5)	41 (15.2–100.6)	26 (15.2–39.9)
5 y PFS (95% CI)	29% (21–39)	43% (33–53)	HR 0.73 (0.52–1.02, *P* = .07)
OS, months, median (95% CI)	57 (37.7–75.1)	85 (42.7–104.3)	61 (44.7–81.7)
5 y OS (95% CI)	49% (39–59)	53% (43–63)	HR 0.87 (0.61–1.26, *P* = .47)
Causes of death			
PCNSL	44/64 (69%)	39/55 (70%)	83/119 (70%)
Complication of Tx	4/64 (6%)	3/55 (5%)	7/119 (6%)
Intercurrent disease	2/64 (3%)	3/55 (5%)	5/119 (4%)
Secondary malignancy^a^	3/64 (5%)	1/55 (2%)	4/119 (3%)
Other	6/64 (9%)	4/55 (7%)	10/119 (9%)
Unknown	5/64 (8%)	5/55 (9%)	10/119 (8%)

Abbreviations: CSF, cerebrospinal fluid; MSKCC, Memorial Sloan Kettering Cancer Center; Tx, treatment; WHO, World Health Organization; LDH, lactate dehydrogenase; R-MBVP, rituximab—methotrexate, BCNU, vincristine, and prednisolone; DLBCL, diffuse large B-cell lymphoma; EFS, event-free survival; PFS, progression free survival; OS, overall survival; CI, confidence interval.

^a^In total 12 s malignancies were reported, 4 of them in patients with relapsed or progressive PCNSL: 2 basocellular carcinoma, 2 melanomas, 2 breast, 2 renal, and 1 each of prostate, colorectal, urothelial, and lung carcinoma.

### Survival Measures

**Figure 1.**
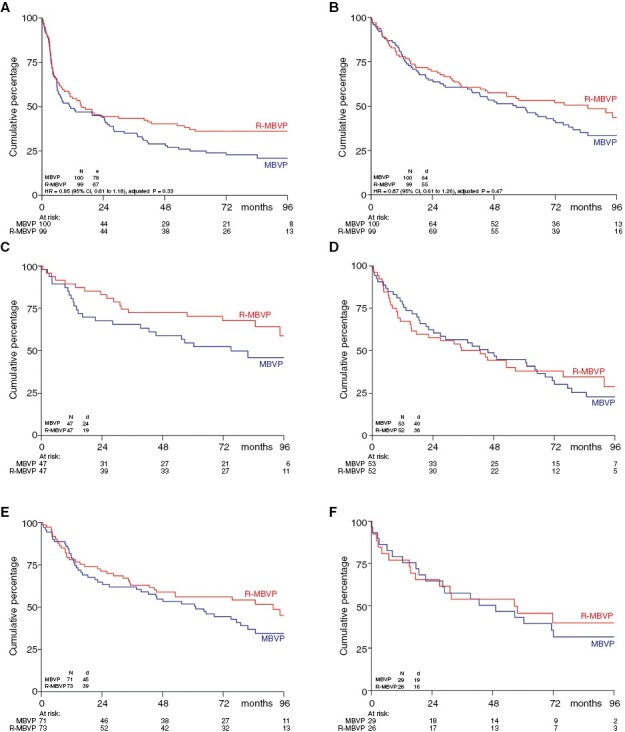
Survival Curves. (A) Event-Free and (B) Overall Survival (C and D) Overall Survival by Age Group (≤60 years, >60 years) and (E and F) WHO Performance Status (0–1, 2–3)

No statistically significant survival differences were found between the 2 arms: for EFS the hazard ratio (HR) for the R-MBVP arm was 0.85, 95% CI 0.61–1.18, *P* = .33; for PFS, HR 0.73, 95% CI 0.52–1.02, *P* = .067; and for OS, HR 0.87, 95% CI 0.61–1.26, *P* = .47. (Figure 1A–C). The 5-year OS after MBVP and R-MBVP was 49% and 53%, respectively (Table 1).

Mortality was 60%: 64/100 patients in the MBVP arm and 55/99 in the R-MBVP arm died, of which 69% as a result of PCNSL and 6% due to treatment toxicity (Table 1). Radiologically defined relapse or progression occurred in 111 patients: 63 in the MBVP arm and 48 in the R-MBVP arm, of whom 95 (86%) died, most (82%) as a result of the lymphoma. The majority of patients (72%) relapsed in the brain parenchyma, 14% in the vitreous and only 4% each in either CSF or systemically (Supplementary Table 2a). Relapses continued to occur throughout follow-up in both arms but less frequently beyond 3 years posttreatment: in year 4, 6 patients relapsed in arm A, of whom 3 in the eye only and 3 in arm B of whom 1 in the eye only. In year 5, 4 patients relapsed in arm A of whom 1 in the eye only, and 3 in arm B, and beyond year 5, 1 patient relapsed in arm A and 2 in arm B (Supplementary Table 2b). All patients with ocular relapse were treated locally only; treatment data for all relapsed patients are given in Supplementary Table 2c.

In total, 88/111 (79%) of relapsed patients received further treatment; median OS time after relapse was 10.5 months, 95% CI 5.9–19.9, and was similar in the 2 arms (HR 0.98, 95% CI 0.6–1.59, *P* = .93).

At multivariable Cox regression analysis, only age > 60 years and dexamethasone dose (as a continuous variable) were independent prognostic factors for EFS, PFS, and OS (Table 2). Treatment arm, performance status, sex, multifocal disease, LDH level, and localizations in CSF, or vitreous fluid were not independently associated with survival outcomes. A post hoc subgroup analysis in younger patients (≤60 years) suggested improved EFS with rituximab (HR 0.48, 95% CI 0.28–0.81) though no statistically significant difference in OS time was found (HR 0.65, 95% CI 0.36–1.19, Figure 1C–D) on the longer term, similar to the results on the short-term.^9^ The 5-year OS of patients < 60 years was 62%.

**Table 2.** Multivariable Analysis for EFS and OS

	EFS		OS	
	HR (95% CI)	*P*	HR (95% CI)	*P*
Rituximab	0.86 (0.61–1.20)	.36	0.84 (0.58–1.23)	.37
Age > 60	2.39 (1.66–3.45)	<.001	2.33 (1.55–3.51)	<.001
WHO > 1	0.69 (0.45–1.05)	.08	1.04 (0.67–1.61)	.88
Sex female	0.79 (0.56–1.13)	.19	0.82 (0.56–1.20)	.32
MMSE ≥ 27/30	0.67 (0.44–1.02)	.06	0.70 (0.45–1.07)	.10
Multifocality	1.42 (0.97–2.09)	.08	0.97 (0.65–1.44)	.87
Vitreous localization	1.13 (0.53–2.41)	.74	1.4 (0.63–3.12)	.41
CSF localization	0.82 (0.55–1.21)	.32	0.89 (0.58–1.38)	.61
Dexamethasone dose (per mg)	1.04 (1.02–1.07)	<.001	1.05 (1.02–1.08)	<.001
LDH elevated	1.16 (0.79–1.71)	.44	1.14 (0.75–1.74)	.55

Abbreviations: EFS, event-free survival; OS, overall survival; HR, hazard ratio; CI, confidence interval; WHO, World Health Organization; MMSE, mini-mental state examination; CSF, cerebrospinal fluid; LDH, lactate dehydrogenase; mg, milligram.

### Neurocognitive Function and Health-Related Quality of Life

Thirty-one and 45 patients were included in the long-term NCF and HRQoL analysis, respectively. Baseline characteristics were similar for patients included in this long-term analysis and the total trial population; only the median age was slightly lower (59 vs. 61 years) and performance status at baseline was slightly higher. Slightly more patients eligible for the long-term analysis of NCF and HRQoL, as compared to the total population, had been treated with rituximab (60–61% vs. 50%), HD-Ara-C (94–98% vs. 81%), and WBRT (56–58% vs. 35%) (Supplementary Table 2). Compliance with NCF and HRQoL testing was ≥60% at all time points, except for NCF at 36 months (55%) (Supplementary Table 3).

**Figure 2.**
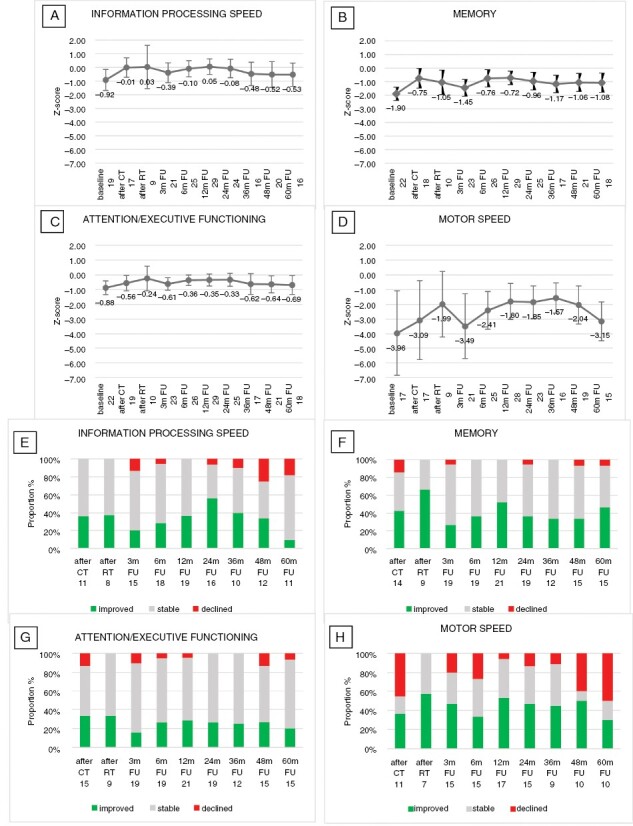
Changes in Neurocognitive Function Over Time. Mean *z*-Scores (95% CI) Over Time for the Neurocognitive Domains (A) Information Processing Speed, (B) Memory, (C) Attention/Executive functioning and (D) Motor speed (Group-Level Analysis). Percentage of Patients with a Clinically Relevant Change (ie Change in *z*-Score > 1) in Neurocognitive Functioning Over Time as Compared to the Baseline Score, (E) Information Processing Speed, (F) Memory, (G) Attention/Executive Functioning, and (H) Motor Speed (Individual Level Analysis). For Each Time Point, the Number of Patients Included in the Analysis is Shown CT, chemotherapy; RT, radiotherapy; *m*, months; FU, follow-up.

For NCF, scores improved after treatment on the group level and, with the exception of motor speed, remained largely stable during the 5 years follow-up period (Figure 2). For motor speed, there was a clinically relevant deterioration after 48–60 months of follow-up. At the individual level most patients retained stable NCF scores over time when compared to their baseline scores or “after chemotherapy” (Supplementary Figures 1 and 3). Of note, for memory, 50% of the patients showed a clinically relevant improvement after 60 months compared to baseline, while for motor speed 47% of the patients showed a clinically relevant deterioration at 60 months.

**Figure 3.**
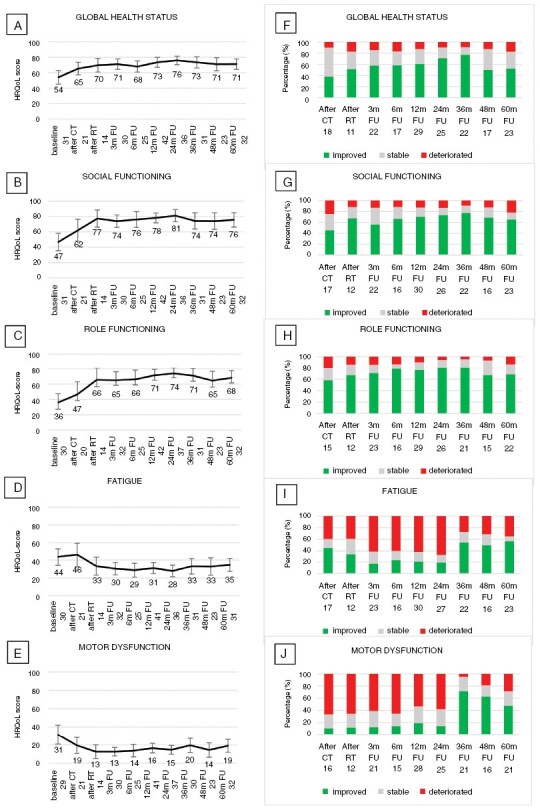
Changes in HRQoL Scores Over Time. Mean (95% CI) HRQoL Scores Over Time for the Scales for (A) Global Health Status, (B) Social Functioning, (C) Role Functioning, (D) Fatigue, and (E) Motor Dysfunction (Group-Level Analysis). Percentage of Patients with a Clinically Relevant Change (ie Change of ≥10 Points) in HRQoL Scores Over Time as Compared to the Baseline Score, Separately for (F) Global Health Status, (G) Social Functioning, (H) Role Functioning, (I) Fatigue, and (J) Motor Dysfunction (Individual Level Analysis). For Each Time Point, the Number of Patients Included in the Analysis is Shown CT, chemotherapy; RT, radiotherapy; *m*, months; FU, follow-up.

For HRQoL, scores at the group level also improved to a clinically relevant extent after treatment, and remained stable thereafter for up to 5 years after treatment, for all preselected scales except fatigue (Figure 3). For fatigue, the improvements after treatment were clinically relevant at all time points except the 60 months assessment. However, fatigue scores remained higher than in norm populations to a clinically relevant extent, ie >10 point difference, at all time points. At an individual patient level, the percentage of patients with stable or improved scores after treatment and up to 60 months of follow-up was relatively stable: for global health status (69–91%), and role and social functioning (81–95% and 78–91%). Results were different for fatigue and motor dysfunction. After 48 and 60 months of follow-up, 31 and 35% of patients showed clinically relevant increased fatigue as compared to baseline. Similarly, for motor dysfunction, a considerable proportion (25–33%) of patients reported more motor dysfunction at 48 and 60 months after treatment than at baseline (Figure 3). For both NCF and HRQoL outcomes, similar results were found in the 18 (NCF) and 25 (HRQoL) irradiated patients available for long-term analysis (Supplementary Figures 2 and 4).

## Discussion

Long-term analyses of this randomized phase 3 study, with a median follow-up of 82 months, confirm our previous findings that rituximab, when added to MBVP chemotherapy, does not improve survival outcomes in patients with PCNSL. This is contradictory to the IELSG32 study, in which PFS and OS were longer in the MATRIx arm (MTX-Ara-C plus rituximab and thiotepa) than in the MTX-Ara-C arm on long-term follow-up, and even the addition of only rituximab prolonged OS compared with MTX-Ara-C alone.^6^ Whether inadvertent differences in the patient populations between the trial arms in these studies explain this discrepancy, or whether the effect of rituximab is more pronounced with less effective chemotherapy (ie HD-MTX-Ara-C only vs. MBVP-HD-Ara-C) remains uncertain. Relapses continued to occur throughout follow-up, as in other studies utilizing WBRT or no consolidation,^3,4,6,7^ but possibly less so in studies with myeloablative chemotherapy followed by ASCT.^5,7^

We found an especially strong prognostic effect of age on survival: for EFS and age > 60 years the HR was 2.39, 95% CI: 1.66–3.45 and for OS the HR was 2.33, 95% CI: 1.55–3.51. This is likely to be at least partially the result of the study design in which patients ≤ 60 years old were consolidated with WBRT whereas older patients received no consolidation other than the HD-Ara-C consolidation prescribed to all patients, because of the known negative effects of WBRT on neurocognitive functioning in elderly patients. Since the results of the second randomization of the IELSG32 trial and the PRECIS trial have been reported, many centers now have adopted myeloablative chemotherapy followed by autologous stem cell transplantation (ASCT) as principle consolidation treatment in fit patients. ASCT, with high-dose thiotepa in the conditioning regimen, is also feasible in fit elderly patients.^25,26^ Whether the apparent greater effect of rituximab on EFS in younger and irradiated patients is a true effect or simply a coincidence remains elusive. However, since no effect was shown on OS, treatment advice cannot be based on this.

We observed no significant and clinically relevant decline for HRQoL or NCF outcomes, except for motor speed, in long-term survivors at the group level. However, at individual patient levels, major variations were found, with stable patients as well as patients who improved or deteriorated to a clinically relevant extent (Figures 2 and 3). The apparent deterioration in motor speed may have been caused by the smaller number of patients in this analysis than in analyses of other cognitive domains—not all centers were equipped with the grooved pegboard test. Additionally, the influence of strength and dexterity cannot be ruled out when assessing motor speed. Other factors, such as disease location, steroid use and treatment delay at diagnosis may also have influenced the results of the NCF and HRQoL analyses but analysis of all these factors for all outcome measures would require correction for multiple testings and much larger patient numbers. Unfortunately, too few irradiated patients had results to allow valid individual longitudinal analysis of long-term NCF and HRQoL outcomes. At the group level, no significant deterioration over time was found but the small number of patients precludes drawing conclusions, for example, regarding the influence of reduced-dose radiotherapy on this apparently preserved NCF in the long term. However, as previously shown, after 2 years of follow-up NCF was shown to be stable even in irradiated patients.^8,27^ As is frequently found in NCF and HRQoL analyses, patients in worse clinical or neurocognitive conditions are less likely to comply with these relatively demanding tests, which may result in an underestimation of neurocognitive deficits in the population under investigation. Nevertheless, deterioration of NCF was found in the WBRT arm of both studies comparing 36–40 Gy WBRT with high-dose chemotherapy and ASCT as consolidation.^6,7^ Two studies investigating NCF after low-dose (23.4 Gy) WBRT found no significant deterioration of NCF until 2–3 years of follow-up.^27,28^ After 3–5 years some deterioration in memory and attention was found in 1 of these series, although this did not differ from patients consolidated with ASCT rather than WBRT.^29^

Interestingly, in our study, scores in all selected HRQoL domains improved to a clinically relevant extent compared to baseline on a group level in the period after treatment and remained stable up to 60 months of follow-up, except for fatigue (where the score after 60 months was slightly below the level of clinical relevance). Nevertheless, compared with the general population the level of functioning in the longer term remains lower, and the level of fatigue, and motor dysfunction is higher.^30^ At the individual patient level, we particularly observed that quite a large proportion of patients report more fatigue than at baseline in the longer term. The persistence of clinically relevant fatigue even years after treatment is a relevant finding for patients with this disease. In the long-term follow-up of other large recent trials on PCNSL fatigue is not mentioned.^6,7^ However, in cancer survivorship, cancer-related fatigue is known to significantly affect physical, emotional, and social well-being and interfere with the ability to maintain daily activities. In systemic NHL severe persistent fatigue has been described to occur in 30–40% of patients 5–20 years after treatment.^31,32^ Similar to our study no association between fatigue and the receipt of rituximab was found at least in the first 24 months of follow-up.^32^

The strength of our study is its prospective, randomized nature with long follow-up including extensive neurocognitive and HRQoL evaluations from baseline onwards. A limitation is the different treatments for patients over and under 60 years old, in our opinion necessitated by the unacceptable risk of neurotoxicity of WBRT in older patients. Because of this, patients over 60 received no consolidation other than the HD-Cytarabine. Although randomization was stratified for age, and these differing treatments were given in both arms, the EFS chosen as the primary outcome measure because of the lack of standard consolidation in older patients, is not easy to compare with other studies. However, with the long-term analysis presented here, OS data are sufficiently mature to exclude a significant effect of rituximab on OS, the most important survival endpoint of treatment, in our study. Regarding NCF and HRQoL outcomes, it is unfortunate that too few results of irradiated patients were available to draw conclusions regarding long-term neurocognitive effects of the reduced-dose WBRT utilized in our study.

In conclusion, long-term analysis confirms that rituximab does not improve outcomes in our study. Neurocognitive functioning or HRQoL outcomes in PCNSL patients receiving (R-) MBVP chemotherapy remain largely stable after treatment up to 60 months of follow-up. At the individual level, most patients’ NCF and HRQoL remain stable or improve, while a minority deteriorate. However, in a significant proportion of patients clinically relevant fatigue persists for years after treatment.

## Supplementary Material

noad224_suppl_Supplementary_Material

## Data Availability

De-identified individual participant data, including a data dictionary defining each field in the concerned data set, will be made available upon reasonable request. Requests for data sharing may be submitted to Jacoline E.C. Bromberg (j.bromberg@erasmusmc.nl).
