# Supplementary material for: Survival, neurocognitive function, and health-related quality of life outcomes after rituximab—methotrexate, BCNU, teniposide, and prednisolone for primary CNS lymphoma: Final results of the HOVON 105/ALLG NHL 24 study
Source: Neuro Oncol. 2023 Dec 1;26(4):724–34. doi: 10.1093/neuonc/noad224 (PMC10995504; doi:10.1093/neuonc/noad224)
Supplement: noad224_suppl_Supplementary_Material [file noad224_suppl_supplementary_material.docx]

**Supplemental**

# Survival, Neurocognitive Function and Health-Related Quality of life after (R-)MBVP for Primary Central nervous System Lymphoma: Final Results of the Randomized Phase III HOVON 105 / ALLG NHL 24 Study

**Supplemental table 1. Neurocognitive Function Tests**

| Function | Test |
| --- | --- |
| Estimation of premorbid IQ | Dutch: NLV (Nederlandse Leestest Volwassenen)  English: National Adult Reading Test |
| Attention/executive function | - WAIS III, digitspan forward and backward  - Trail Making Test parts A and B  - Letter Digit Substitution Test (oral presentation) |
| Verbal memory | Rey Auditory Verbal Learning test |
| Motor | Grooved Pegboard Test |

**Supplemental Table 2a. Site of first relapse (all relapses)**

|  | MBVP (n=63) | R-MBVP (n=48) | Total(N=111) |
| --- | --- | --- | --- |
| CSF | 3 (5%) | 1 (2%) | 4 (4%) |
| brain parenchyma | 46 (73%) | 34 (71%) | 80 (72%) |
| vitreous fluid | 6 (10%) | 9 (19%) | 15 (14%) |
| systemic | 3 (5%) | 1 (2%) | 4 (4%) |
| Unknown/other | 5 (8%) | 3 (6%) | 8 (7%) |

**Supplemental Tabel 2b. Site of first relapse occurring after year 3:**

|  | MBVP (n=11) | R-MBVP (n=8) | Total (N=19) |
| --- | --- | --- | --- |
| CSF |  |  |  |
| brain parenchyma | 5 | 7 | 12 |
| vitreous fluid | 4 | 1 | 5 |
| systemic | 2 |  | 2 |
| Unknown/other |  |  |  |

**Supplemental table 2c. treatments at relapse**

|  | MBVP | R-MBVP | Total |
| --- | --- | --- | --- |
| Ocular RT | 1 | 1 | 2 |
| Ocular MTX and/or rituximab | 4 | 5 | 9 |
| Intra-CSF MTX and/or Ara-C | 4 | 3 | 7 |
| Systemic treatments: |  |  |  |
| Temozolomide | 5 | 5 | 10 |
| Lenalidomide | 1 | 2 | 3 |
| Ibrutinib |  | 1 | 1 |
| CYVE | 1 | 2 | 3 |
| HD-MTX / MBVP | 2 | 7 | 9 |
| HD-MTX and ASCT | 4 |  | 4 |
| ASCT other | 4 | 1 | 5 |
| Systemic other | 2 | 2 | 4 |
| WBRT | 13 | 12 | 25 |
| none | 11 | 12 | 23 |
|  |  |  |  |

**Supplemental Table 3. Clinical features for those who participated in long-term neuropsychological evaluation (NPE) and health-related quality of life (HRQoL) assessment, compared to the total study population.**

|  | Eligible for long-term analysis | |  |
| --- | --- | --- | --- |
|  | **NPE**  **n=31** | **HRQoL**  **n=45** | **Whole study population**  **n=199** |
| Sex (n, % male) | 12 (39%) | 24 (53%) | 109 (55%) |
| Age (median, IQR) | 59 (57-62) | 59 (57-63) | 61 (55-67) |
| WHO performance score (n, %)  WHO 0  WHO 1  WHO 2  WHO 3 | 11 (35%)  14 (45%)  3 (10%)  3 (10%) | 14 (31%)  23 (51%)  4 (9%)  4 (9%) | 43 (22%)  101 (51%)  34 (17%)  21 (10%) |
| Comorbidities active at baseline (n, % ≥2)  Missing | 15 (48%)  0 | 22 (49%)  0 | 104 (52%)  0 |
| Solitary lesion (n, %)  Missing/ NA | 16 (52%)  0 | 25 (56%)  1 (2%) | 103 (52%)  19 (10%) |
| Bilateral involvement (n, %)  Missing/ NA | 12 (39%)  0 | 15 (33%)  1 (2%) | 75 (38%)  19 (10%) |
| Deep structures involved (n, %)  Missing/ NA | 21 (68%)  0 | 29 (64%)  1 (2%) | 125 (63%)  13 (7%) |
| Treatment | | | |
| Rituximab | 19 (61%) | 27 (60%) | 99 (50%) |
| HD Ara-C (n, %) | 29 (94%) | 44 (98%) | 161 (81%) |
| WBRT (n, %) | 18 (58%) | 25 (56%) | 70 (35%) |
| Radiation boost given (n, %) | 12 (39%) | 15 (33%) | 39 (20%) |
| Intrathecal treatment given (n, %) | 1 (3%) | 4 (9%) | 16 (8%) |

IQR = interquartile range, WHO = world health organization, NA = not available, HD Ara-C = high-dose cytarabine, WBRT = whole brain radiotherapy.

**Supplemental Table 4.** **Compliance with neuropsychological evaluation (NPE) and health-related quality of life (HRQoL) assessment for those patients included in the long-term analysis.**

| NPE | Baseline | After CT | After RT | 3m | 6m | 12m | 24m | 36m | 48m | 60m |
| --- | --- | --- | --- | --- | --- | --- | --- | --- | --- | --- |
| Observed | 23 | 19 | 10 | 23 | 26 | 29 | 25 | 17 | 21 | 18 |
| Expected | 31 | 30 | 18 | 31 | 31 | 31 | 31 | 31 | 30 | 30 |
| Compliance rate | 74% | 63% | 55% | 74% | 84% | 93% | 81% | 55% | 70% | 60% |
|  |  |  |  |  |  |  |  |  |  |  |
| HRQoL assessment | **Baseline** | **After CT** | **After RT** | **3m** | **6m** | **12m** | **24m** | **36m** | **48m** | **60m** |
| Observed | 31 | 21 | 14 | 32 | 25 | 42 | 37 | 31 | 23 | 32 |
| Expected | 45 | 45 | 25 | 44 | 44 | 44 | 42 | 40 | 37 | 36 |
| Compliance rate | 69% | 47% | 56% | 73% | 57% | 95% | 88% | 78% | 62% | 89% |

B

D

C

A

**Supplemental Figure 1. Percentage of patients with a clinically relevant change (i.e. change in z-score >1) in neurocognitive functioning over time as compared to ‘after chemo’ score, separately for (A) information processing speed, (B) memory, (C) attention / executive functioning, and (D) motor speed.**  For each time period, the number of patients included in the analysis are shown.

CT = chemotherapy, RT = radiotherapy, m = months, FU = follow-up.

D

B

C

A

**Supplemental Figure 2. Mean z-scores (95% confidence interval) over time for the neurocognitive domains (A) information processing speed, (B) memory, (C) attention / executive functioning and (D) motor speed for those patients who received irradiation (group level analysis).** For each time point, the number of patients included in the analysis are shown.

CT = chemotherapy, RT = radiotherapy, m = months, FU = follow-up.

A

B

C

D

E

**Supplemental Figure 3. Percentage of patients with a clinically relevant change (i.e. change in mean score >10) in health-related quality of life over time as compared to ‘after chemo’ score, for the scales (A) global health status, (B) social functioning, (C) role functioning, (D) fatigue and (E) motor dysfunction.** For each time point, the number of patients included in the analysis are shown.

CT = chemotherapy, RT = radiotherapy, m = months, FU = follow-up

D

C

A

B

**Supplemental Figure 4. Mean (95% confidence interval) HRQoL scores over time for the scales (A) global health status, (B) social functioning, (C) role functioning, (D) fatigue and (E) motor dysfunction for those patients who received irradiation (group analysis).** For each time point, the number of patients included in the analysis are shown.

CT = chemotherapy, RT = radiotherapy, m = months, FU = follow-up

E
